# Valorization of Potato Peels (*Solanum tuberosum*) Using Infrared-Assisted Extraction: A Novel Sprouting Suppressant and Antibacterial Agent

**DOI:** 10.3390/foods13213445

**Published:** 2024-10-28

**Authors:** Layan Helmi, Alissar Al Khatib, Hiba N. Rajha, Espérance Debs, Adla Jammoul, Nicolas Louka, Nada El Darra

**Affiliations:** 1 Faculty of Heath Sciences, Beirut Arab University, Tarik El Jedidah, Riad EL Solh, P.O. Box 115020, Beirut 1107 2809, Lebanon; l.ahelmi@bau.edu.lb; 2 Department of Nursing, Faculty of Health Sciences, Almoosa College, Al Ahsa P.O. Box 5098, Saudi Arabia; a.khatib@almoosacollege.edu.sa; 3 Unité de Recherche Technologies et Valorisation Agro-Alimentaire, Centre d’Analyses et de Recherche, Faculté des Sciences, Université Saint-Joseph de Beyrouth, Riad El Solh, P.O. Box 17-5208, Beirut 1104 2020, Lebanon; hiba.rajha@usj.edu.lb (H.N.R.); nicolas.louka@usj.edu.lb (N.L.); 4 Department of Biology, Faculty of Arts and Sciences, University of Balamand, Tripoli P.O. Box 100, Lebanon; esperance.debs@balamand.edu.lb; 5 Food Department, Lebanese Agricultural Research Institute, P.O. Box 2611, Beirut 1107 2809, Lebanon; ajammoul@lari.gov.lb

**Keywords:** potato peels, water bath extraction, infrared-assisted extraction, antibacterial activity, sprouting suppressant, agricultural practices, waste management

## Abstract

Recently, there has been a growing interest in reducing waste to promote environmental sustainability, with particular focus on agricultural by-products, especially fruits and vegetables. Potato, a widely used crop across various industries, generates a significant amount of peel waste. This study aims to valorize potato peels using water bath extraction (WBE) and infrared-assisted extraction (IRAE), both with distilled water as the solvent, followed by assessments of antioxidant, antibacterial, and anti-sprouting activities. Optimization using response surface methodology identified optimal extraction conditions for WBE (90 °C for 70 min) and IRAE (80 °C for 10 min), with both methods yielding 3.5 mg GAE/g DM in polyphenol content. IRAE demonstrated superior energy efficiency and enhanced antioxidant activity. The extracts exhibited antibacterial properties against both Gram-positive (*Listeria monocytogenes*) and Gram-negative bacteria (*Proteus* sp. and *Salmonella* sp.), with inhibition zones ranging from 10 to 14 mm. Furthermore, the potato peels extract showed significant anti-sprouting effects at room temperature, reducing both the number and size of sprouts compared with the control. HPLC analysis showed the presence of different phenolic compounds such as rutin, catechin, caffeic acid, protocatechuic acid, chlorogenic acid, *p*-coumaric acid, and gallic acid in one or both extracts. These findings suggest that potato peels extract holds potential for applications in the food industry as a natural preservative due to its antioxidant properties, as well as a sprout suppressant. The antibacterial activity of the extracts suggests their potential as a natural preservative as well, offering protection against both Gram-positive and Gram-negative bacteria that may be present in food.

## 1. Introduction

In recent years, “food waste” has garnered significant attention due to its contribution to environmental pollution. After processing, food waste has been identified as a valuable source of proteins, fats, carbohydrates, and various other nutritional components [1]. Vegetable waste, in particular, holds potential as a source of natural flavors and antioxidants, with applications extending to the pharmaceutical and cosmetic industries [2]. Among widely cultivated crops, potato (*Solanum tuberosum*) ranks as the fourth largest, following rice, wheat, and maize, and is the most significant non-cereal crop worldwide [3]. Potato peels, one of the largest food by-products, especially from the potato chips and frozen French fries industries, have attracted growing interest for their potential reuse and valorization.

The extraction process is a critical step in recovering bioactive compounds from both products and by-products, facilitating their valorization while maintaining their biological activities. Several extraction technologies exist, with water bath extraction (WBE) being one of the simplest and most widely used methods. This technique relies on diffusion and osmosis, where the solid matrix releases its chemical substances into the liquid phase [4]. Notably, distilled water has been identified as an effective solvent for maximizing phenolic compound extraction, outperforming solvents such as hexane, chloroform, and ethyl acetate [5]. As methanol is toxic, using safer alternatives like distilled water, which is non-toxic, inexpensive, and environmentally friendly, is highly advantageous for human consumption and sustainable extraction practices [6].

Among the emerging innovative techniques, ultrasound-assisted extraction (UAE) has gained attention for its reduced solvent usage compared with WBE, offering high reproducibility, greater extraction efficiency, and shorter extraction times, while also yielding extracts with higher purity [7]. This method involves the emission of ultrasonic waves through devices such as probes and baths [8]. Other emerging extraction technologies include the intensification of vaporization by decompression to the vacuum (IVDV) [9], electric field techniques [10], high voltage electric discharge (HVED) [11], β-cyclodextrin-assisted extraction [12], and the use of deep eutectic solvents [13]. Infrared-assisted extraction (IRAE) is another promising technique, utilizing infrared irradiation—an electromagnetic wave with high permeability and low energy consumption—which enhances its appeal for research applications [14]. A study on IRAE for polyphenol extraction from apricot pomace demonstrated a maximal yield under the same energy input and treatment duration when compared with UAE and WBE [15]. Furthermore, IRAE has been applied to various by-products and matrices, such as blood orange peels [16], *Eryngium creticum* leaves [17], purple corn cobs [18], pomegranate peels [19], orange peels [20], and olive leaves [20]. To our knowledge, this technique has not yet been explored for potato peels extraction [21].

In addition to its antiradical properties, potato peels extract exhibits antioxidant and antimicrobial effects [22]. Methanolic potato peels extract showed a significant inhibition effect against two Gram-positive bacteria, *Bacillus amyloliquefaciens* and *Staphylococcus aureus*, and against two Gram-negative bacteria, *Escherichia coli* and *Pseudomonas aeruginosa*.

On another level, potato tuber sprouting is a common phenomenon occurring in stored potatoes. The most widely used sprout inhibitor has been CIPC (Isopropyl N-(3-chlorophenyl) carbamate), an herbicide introduced in 1951, typically applied at temperatures of 8–12 °C for optimal efficacy [23]. However, in January 2020 the European Food Safety Authority (EFSA) banned the use of CIPC in the EU due to concerns over its toxicity and carcinogenic potential [24]. This has prompted the search for alternative methods to inhibit sprouting. In a previous study, essential oils of garlic, rosemary, thymus, and palmarosa were evaluated as potential sprout inhibitors at room temperature (RT). These oils, applied in vapor form for 24 h with the potatoes placed in wooden boxes and the oils dispersed on filter paper, were observed to inhibit sprouting over a 30-week period [25]. Essential oils have been shown to inhibit potato sprouting by altering levels of accumulated reducing sugars, ethylene production, and the expression of genes involved in the sprouting process [26].

Based on the above, this study aims to extract bioactive compounds from potato peels using water bath extraction (WBE) and infrared-assisted extraction (IRAE), with distilled water as the solvent. The optimization of the extraction process will be conducted using response surface methodology. Following extraction, the crude extracts will be evaluated for their antioxidant, antiradical, antimicrobial, and anti-sprouting activities.

## 2. Materials and Methods

### 2.1. Raw Material

The potato peels were acquired from the Master Chips company in Beqaa, Lebanon, and were of the Fontane and Rosetta varieties. The peels were placed in a hot air oven at 45 °C for 24 h. After drying, they were stored in the fridge until ready for extraction.

### 2.2. Dry Matter Content

The dry matter content was determined by weighing 5 g of the potato peels, which were then dried for 24 h at 105 °C in a hot air oven. After drying, the peels were reweighed [27]. The dry matter (DM) content of initial raw material was found to be 89 ± 0.2%.

### 2.3. Chemicals

All reagents used were of analytical grade. Folin–Ciocalteu, DPPH (2,2-Diphenyl-picrylhydrazyl), sodium carbonate, Trolox solution (6-hydroxy-2,5,7,8-tetramethyl-chroman-2-carboxylic acid), and gallic acid were provided by SIGMA Aldrich (Steinheim, Germany). Mueller Hinton Agar was provided by Condalab (Madrid, Spain). Mueller Hinton Broth was provided by HIMEDIA (Mumbai, India). Nutrient agar was provided by OXOID (Hampshire, UK).

### 2.4. Extraction Techniques

#### 2.4.1. Water Bath Extraction

Water bath extraction was conducted using a shaking water bath (DKZ series, Shaking Water Bath) operating at 660 W and a frequency of 50 Hz at various times and temperatures, as outlined in Table 1. Potato peels were placed in beakers containing pre-heated distilled water, covered with aluminum foil, and immersed in the water bath at the specified temperature for the designated duration. The extraction ratio was set at 1 g of peels to 10 mL of distilled water [28]. Following extraction, the mixture was centrifuged at 4500 rpm for 15 min, and the supernatant was collected and stored in the freezer for further analysis.

#### 2.4.2. Infrared-Assisted Extraction

**Figure 1:**
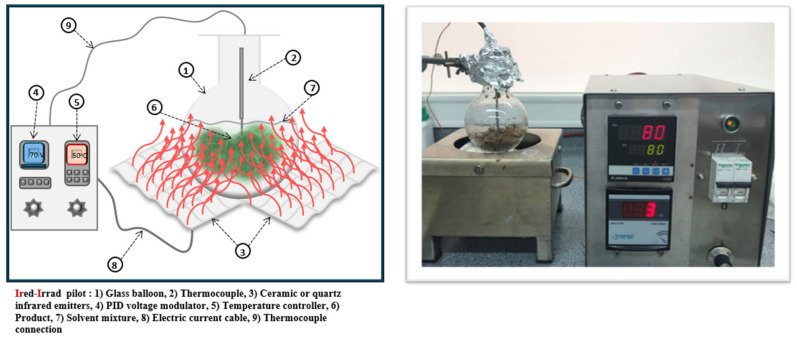
Infrared-assisted extraction system: schematic and experimental setup.

The apparatus *Ired-Irrad*^®^ was used for the infrared-assisted extraction (patent 2017/11-11296L) of potato peels. It consists of a ceramic infrared emitter that is connected to a proportional–integral–derivative (PID) control and temperature adjustment system (Figure 1). Pre-heated distilled water was placed in a round bottom flask with the peels then added in the flask. The flask was placed around 10 mm away from the ceramic emitter. The solid to liquid ratio adopted was 1 g in 10 mL distilled water. After extraction, the extract was centrifuged for 15 min at 4500 rpm, and the supernatant collected was stored in the freezer until further use. Various times and temperatures were tested, as outlined in Table 1.

### 2.5. Total Phenolic Content (TPC)

The method for TPC was conducted using the Folin–Ciocalteu method [11,29]. An amount of 0.2 mL of the gallic acid standard or sample was mixed with 1 mL of Folin–Ciocalteu reagent to prepare the samples and standard curve. After one minute, 0.8 mL of sodium carbonate solution (7.5%) was added and mixed by vortex. The test tubes were placed in a water bath for 10 min at 60 °C, then light absorption was read using a UV–Vis spectrophotometer at 750 nm. The blank was prepared as stated before with distilled water instead of the sample. Total phenolic content was expressed in mg gallic acid equivalent per gram dry matter (mg GAE/g DM).

### 2.6. DPPH Scavenging Assay

The DPPH scavenging assay method is based on the color change in DPPH solution from purple to yellow. As discoloration increases (to a more yellow color), this indicates that there is a better scavenging capacity of the extract [30]. The method is as follows: Dissolve 3.94 mg of DPPH powder in 100 mL methanol (0.1 mM DPPH solution). Then, 4 mL of the DPPH solution is mixed with 0.2 mL of the extract and placed in the dark for 30 min. For the control, 0.2 mL of distilled water is mixed with the DPPH solution. Using the spectrophotometer at 517 nm, the blank used is pure methanol. This is a slightly modified method stated in another study [31]. After measuring the light absorbance of the samples at 517 nm, the percentage inhibition can be calculated using the following equation:

$$\frac{Absorbance\;Control-Absorbance\;Extract}{Absorbance\;Control}\times 100$$

In addition to calculating the percentage inhibition by performing the DPPH test, a Trolox calibration curve was carried out to express the antiradical activity as a function of Trolox concentration. Trolox solution (6-hydroxy-2,5,7,8-tetramethyl-chroman-2-carboxylic acid) was diluted in methanol. After calculating the percentage inhibition for each sample, the results were converted to concentration for Trolox eq according to the calibration curve. Results are expressed in µg Trolox equivalent per mL (µg TE/mL).

### 2.7. Experimental Design for WBE and IRAE Optimization

Response surface methodology (RSM) with a central composite design was used to optimize both water bath and infrared-assisted extractions in terms of TPC and antioxidant activity DPPH. A rotatable central composite design (2^2^ + stars) was made to study the effect of the two independent factors (time and temperature) in 12 runs (four factorial points, four star points, and four center points) on the studied responses: TPC and antioxidant activity. The time and temperature varied between five levels (−α, −1, 0, +1, +α) from 18.7 min to 61.3 min, and from 45.8 °C to 74.2 °C, respectively.

### 2.8. Antimicrobial Testing

The antimicrobial activity of both extracts, using water bath and infrared irradiation, were assessed against three Gram-positive (*Listeria monocytogenes*, *Bacillus* sp., and *Staphylococcus aureus*) and six Gram-negative bacterial strains (*Escherichia coli*, *Klebsiella pneumoniae*, *Pseudomonas aeruginosa*, *Salmonella* sp., *Proteus* sp., and *Shigella* sp.). The bacterial strains used were provided by the Department of Health Sciences at Beirut Arab University and the American University of Beirut Medical Center. The cultures of bacteria were kept in their suitable agar slants at 4 °C throughout the study and used as stock bacterial cultures.

The antibacterial effect of potato extracts was assessed using the agar disk diffusion method, and the minimum inhibitory concentration (MIC) was performed and determined using the broth dilution method.

After performing the response surface methodology, the extracts obtained under optimal conditions were selected to perform microbiological activity. However, after initial screening of the extract’s ratio of 1 g to 10 mL distilled water, it was found that the effect was not significant. Therefore, to concentrate the extracts, 1 g in 5 mL distilled water was performed for the water bath and infrared extractions [32].

#### 2.8.1. Inoculum Standardization

Bacterial inoculums were obtained from the stock cultures and inoculated in Lysogeny broth (LB) then incubated at 37 °C for 18–24 h. From the freshly grown cultures, decimal dilutions were made in 0.9% sterile saline until reaching a turbidity of 0.5 McFarland (10^8^ CFU/mL), for testing the antibacterial effects of the extracts [33].

#### 2.8.2. Agar Disk Diffusion

For the microbiological activity, the disk diffusion method was performed to test the ability of the extract to inhibit bacterial growth. In brief, bacterial suspension of 10^8^ CFU/mL was streaked on the surface of Mueller Hinton Agar (MHA) using a sterile cotton swab. After 15 min, sterilized 5 mm filter paper disks were placed on the agar using forceps that were heat sterilized. Then, 20 µL of the extracts (sterilized by filtration through a 0.45 μm membrane filter) was pipetted on their respective disks [34]. Filter paper disks with distilled water were used as the negative control, while Gentamicin disks (10 μg/disk) were used as the positive control. The plates were left at RT around an hour before incubation at 37 °C for 24 h. After overnight incubation, the inhibition zones were measured in mm. The method was performed in duplicate.

#### 2.8.3. Minimum Inhibitory Concentration

The minimum inhibitory concentration (MIC) is the lowest concentration of potato peels extract that inhibits the visible growth of the tested bacterial strains. The MICs of water bath and infrared extracts that showed promising inhibition in the agar disk diffusion method against the nine bacterial strains were determined.

On a 96-well microtiter plate, the MICs of the optimum extracts WBE and IRAE for each bacterial strain were studied. A volume of 100 µL of Mueller Hinton Broth (MHB) was added to all the wells (1–12). An amount of 100 µL of the extract was added in the first well followed by two-fold serial dilution in the first ten wells, where 10 µL of the bacterial suspension (10^5^ CFU/mL) was added. Well 11 served as a positive control. In well 12, 10 µL of tested standardized inoculum were added, which served as a negative control. After an overnight incubation at 37 °C for 24 h, the microtiter plates of each potato peels extract were read to determine the MIC that inhibited the visual bacterial growth [35].

### 2.9. Anti-Sprouting Protocol

Organic potatoes were purchased from a local store in Beirut, Lebanon. They were split into two groups: the experimental group (exposed to the extract) and the control group (exposed to tap water). The potatoes were washed to remove any impurities then blot dried using tissue paper. Potatoes for each group were weighed before and after treatment. In two separate plates, tap water was placed in one of them and the extract placed in the other. Potatoes of each group were placed on the plates and moved around until all sides were covered with the liquid. The process was repeated, then potatoes were weighed. The sprout number and sprout size were recorded for each potato in every group. 

### 2.10. High Performance Liquid Chromatography (HPLC) Analysis

The phenolic compounds in the extracts obtained from potato peels by the IRAE and WB methods in their optimal conditions were analyzed using high-performance liquid chromatography (HPLC) to quantify and identify. The HPLC system used for this analysis was an Agilent 1100 Series system (Teknokroma Professional Friendly Lichrospher 100 RP18 5 mM, 25 × 0.46, serial number NF-21378, Barcelona, Spain) equipped with an autosampler, a Zorbax column oven (Barcelona, Spain), and a diode array detector. A C18 column (25 × 0.46 mm) was employed to separate the phenolic compounds. The standards used for identification and quantification were gallic acid, protocatechuic acid, hydroxybenzoic acid, catechin, chlorogenic acid, caffeic acid, *p*-coumaric acid, rutin, ellagic acid, trans-cinnamic acid, and quercetin. The mobile phase consisted of acidified nanopure water at pH 2.3 with HCl (A) and methanol (B) of HPLC grade. The elution process was performed under isocratic conditions, starting with 85% A and 15% B from 0 to 5 min. A gradient profile was then applied from 5 to 30 min, transitioning from 85% A and 15% B to 0% A and 100% B followed by isocratic conditions from 30 to 35 min with 0% A and 100% B. The injection volume was 10 µL and the flow rate was set at 1 mL/min [36]. The identification of phenolic compounds was based on comparing the retention times of the detected peaks with those of the original standard compounds. The concentration of phenolic compounds was determined by constructing standard curves for each individual compound using different concentrations of the corresponding standards.

### 2.11. Statistical Analysis

Analyses were performed using IBM SPSS Statistics 27 (SPSS Inc., Chicago, IL, USA). Data were analyzed using a one-way ANOVA and post hoc with the significant differences being at the 95% confidence interval.

For the optimization part using RSM, results were statistically achieved using STATGRAPHICS Centurion XVI.I (Statgraphics 18, The Plains, VA, USA).

## 3. Results and Discussion

### 3.1. Effect of Time and Temperature on TPC and DPPH

Response surface methodology was conducted with the aim of determining and investigating the optimal conditions for the highest phenolic concentrations in water bath and infrared extracts.

The impact of time and temperature on TPC and antiradical activity was analyzed according to the Pareto chart and 3D mesh for both the WB and IR extracts. Table 1 summarizes the results of all the runs performed.

**Table 1:** Response surface methodology central composite design for time and temperature and the experimental responses TPC and DPPH of potato peels WBE and IRAE.

	Run	Temperature (T) °C	Time (t) min	WBE TPC mg GAE/g DM	WBE-DPPH TE μg/mL	IRAE TPC mg GAE/g DM	IRAE- DPPH TE μg/mL
Factorial Points	1	45.8 (−1)	18.7 (−1)	1.44 ± 0.13	38 ± 1.5	1.2 ± 0.1	22 ± 2
	2	74.2 (+1)	18.7 (−1)	1.71 ± 0.2	118 ± 2.1	2.49 ± 0.2	150 ± 3.1
	3	45.8 (−1)	61.3 (+1)	1.78 ± 0.2	54 ± 2	1.95 ± 0.12	31 ± 2.04
	4	74.2 (+1)	61.3 (+1)	2.79 ± 0.17	132 ± 2.2	2.05 ± 0.1	145 ± 3.15
Star Points	5	40 (−α)	40 (0)	1.29 ± 0.1	31 ± 1.7	1.2 ± 0.11	22 ± 1.1
	6	80 (+α)	40 (0)	2.61 ± 0.12	126 ± 2.9	2.54 ± 0.21	151 ± 4.01
	7	60 (0)	10 (−α)	1.39 ± 0.15	36 ± 0.8	1.27 ± 0.13	20 ± 1.2
	8	60 (0)	70 (+α)	2.10 ± 0.2	47 ± 0.9	1.78 ± 0.11	32 ± 2.01
Center Points	9	60 (0)	40 (0)	2.03 ± 0.15	43 ± 0.9	1.93 ± 0.07	47 ± 3.1
	10	60 (0)	40 (0)	2.03 ± 0.13	43 ± 1.1	2.03 ± 0.02	61 ± 2.2
	11	60 (0)	40 (0)	1.83 ± 0.13	43 ± 1.2	1.64 ± 0.05	40 ± 1.5
	12	60 (0)	40 (0)	2.05 ± 0.14	43 ± 1.1	1.81 ± 0.1	43 ± 2.4

**Figure 2:**
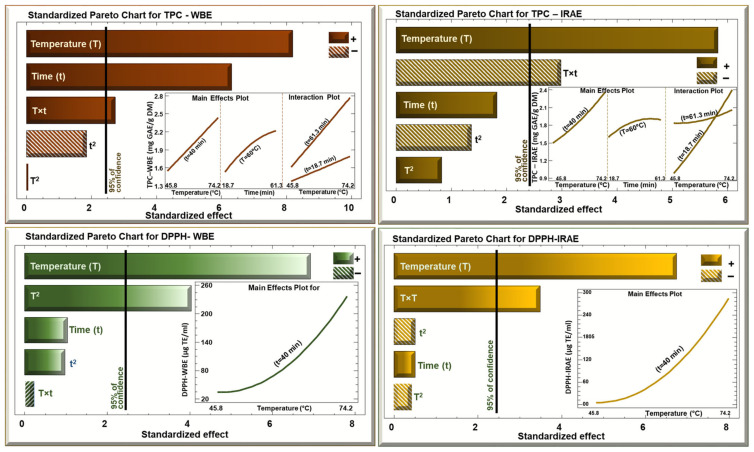
Standardized Pareto charts for TPC and DPPH of potato peels WBE and IRAE. Bars that do not cross the vertical line are considered statistically insignificant.

In the Pareto charts (Figure 2) with WBE, temperature and time had a significant (*p* < 0.01) linear positive effect on the extraction of polyphenols from potato peels. The interaction between time and temperature also had a positive effect, however, it is less significant than with time or temperature alone. As for the quadratic effects of time (tt) and temperature (TT), there were no significant effects. Regarding IRAE, temperature had a significant positive linear effect on TPC and the interaction between time and temperature also had a significant negative effect. Time and the quadratic effects of time and temperature were not significant in this case. The Pareto chart for the Trolox equivalent results shows similar values for both WBE and IRAE. Temperature and the quadratic effect of temperature have a positive significant effect (*p* < 0.01). This means that as temperature increases, the Trolox equivalent increases, indicating a higher antiradical activity (since DPPH percentage increases). When going from −1 (45.8) to +1 (74.4), the Trolox equivalent in WBE increases from 30 to 230 Trolox eq microg/mL while the time is set in the middle level at 40 min and the slope is a parabolic shape indicating that the increase is becoming more important as temperature increases. Time and the quadratic effect of time were not significant in this case. The difference between the WBE and IRAE was that the maximum level of Trolox equivalent reached in IRAE (285 μg/mL) was more than that reached in WBE (230 μg/mL). This means that the extracts in the case of IRAE possess a higher antiradical effect than the extracts obtained by water bath.

**Figure 3:**
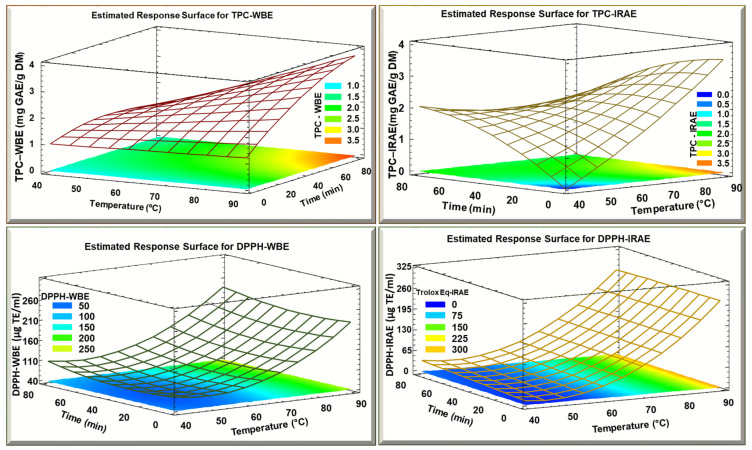
Estimated response surface for TPC and DPPH of potato peels WBE and IRAE as a function of time and temperature.

The 3D model (Figure 3) gives a general idea concerning the evolution of the response parameters (TPC WBE and TPC IRAE) as a function of both time and temperature. To maximize TPC in WBE (3.5 mg GAE/g DM), it required maximal time (70 min) and temperature (90 °C), while for IRAE, the maximal TPC (3.5 mg GAE/g DM) was obtained at high temperature (90 °C) but for a short time of around 20 min (the blue shaded part). Hence. The extraction was conducted rapidly, suggesting a low consumption of energy for this process. When prolonging the treatment time, this led to a negative effect by causing degradation of the phenolic compounds (indicated by a lower TPC result). The advantage of IRAE is that it results in a high TPC during a short time due to its ability to induce significant cellular and structural modifications. The molecular vibrations caused by infrared irradiation facilitate the release of polyphenols from plant cells [37]. In the case of the Trolox equivalent results, as seen before in the Pareto charts, time did not have a significant effect, only temperature had a significant effect. So, whatever the temperature, time had no effect in both cases of WBE and IRAE. The difference was that the maximum reached at WBE (250 μg/mL) was less than the maximum reached while using IRAE (300 μg/mL).

Hence, temperature had a positive effect on polyphenol extraction; indeed, heightening the temperature can lower surface tension and viscosity, thus promoting diffusion which enhances extraction [38]. A similar study reported that higher temperatures positively influenced the extraction of polyphenols from potato peels, provided that the temperature did not exceed the threshold at which polyphenols degrade [39].

### 3.2. Optimization

Response surface methodology allows the optimization of response parameters (TPC and DPPH) and generates a model (second-degree equations) predicting the variation of TPC and DPPH as a function of time and temperature (operational parameters).

The regression equations which have been fitted to the data, along with R^2^ for each test, are shown in Table 2. The values of the variables are as specified in their original units.

**Figure 4:**
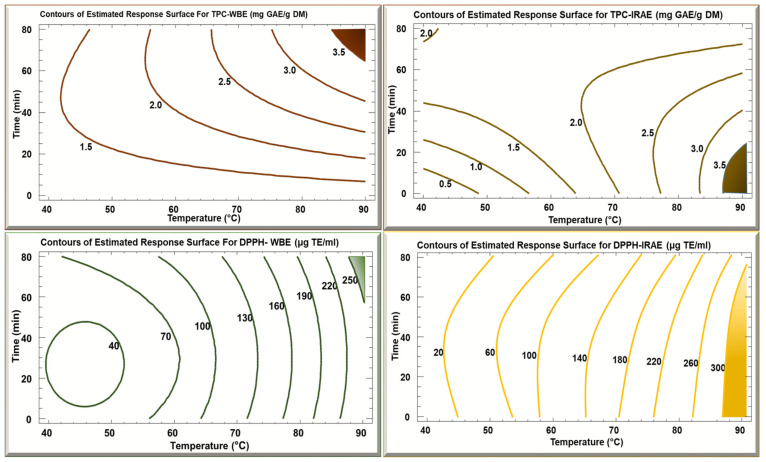
Contours of estimated response surface for TPC and DPPH of potato peels WBE and IRAE as a function of time and temperature.

Figure 4 shows the contours of the estimated response surfaces of TPC and DPPH as a function of time and temperature. The maximum TPC yield of 3.5 mg GAE/g DM, in the case of WBE, was obtained at a high temperature of 90 °C during 70 min. As for the IRAE, the maximum yield of 3.5 mg GAE/g DM according to the figure was also at 90 °C for 20 min. However, when conducting trials to confirm the results, it was found that at 80 °C for 10 min it gave the higher yield of 3.5 mg GAE/g DM. The Trolox eq. in both cases of WBE and IRAE were consistent with the TPC results. The optimum in the case of WBE was 250 Trolox equivalent at 90 °C for 70 min, while for IRAE at 80 °C for 10 min it was over 300 Trolox eq. In the case of IRAE, the TPC and Trolox eq. yield were higher than that of WBE. This can be related to the different mechanisms of action of the extraction methods and their efficiency in recovering the bioactive compounds. For IRAE, its high heat transfer capacity and penetration capacity, as well as the irradiation exciting the sample molecules by twisting, bending, and stretching is related to better extraction of compounds [40], while with WBE, the extraction requires a longer time before heat is conducted and transferred to the matrix [41].

**Figure 5:**
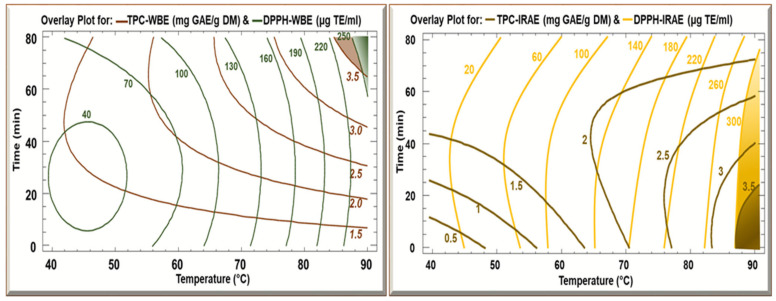
Overlay plots for TPC and DPPH of potato peels WBE and IRAE as a function of time and temperature.

In the overlay plots (Figure 5), it is shown that the optimal zone in the case of WBE is at maximal time (70 min) and temperature (90 °C), while in IRAE the optimal zone is at the high temperature (80 °C) but with a shorter time of 10 min.

The overlay plots further confirm the abovementioned observation. Optimal conditions reached were 90 °C for 70 min in the case of WBE and 80 °C for 10 min in the case of IRAE. Regarding the exposure to high temperatures of 80 and 90 °C without polyphenol destruction, this could be related to the type of polyphenols since the most abundant polyphenols present in the potato peels are chlorogenic acid and caffeic acid. In our findings, the extracts presented chlorogenic acid and/or caffeic acid, as further discussed in the HPLC section. It was found that chlorogenic acid, extracted from eggplant, can withstand a high temperature without degradation until it reaches 250 °C [42]. Another study extracting polyphenols from coffee beans showed that chlorogenic acid and caffeic acid had a high thermostability when the temperature exceeded 100 °C and the extraction process was still running. This was linked to the melting points of these polyphenols where chlorogenic acid has a melting point at 207 °C while caffeic acid has a melting point at 223 °C. However, when exposed to longer periods of time, the chlorogenic amount began to decompose when exposed to a high temperature of 220 °C for 40 min [43].

After performing the experiment at the optimal conditions (90 °C for 70 min for WBE and 80 °C for 10 min for IRAE), the TPC and Trolox equivalent results were similar. A study evaluated the difference between WBE, UAE (bath), and IRAE on polyphenol extraction from *Saussurea lappa* and similar results were obtained, where RSM results concluded that IRAE requires less time compared with the other extraction techniques, while also yielding a high polyphenol content [44]. This is further confirmed in another study conducted comparing WBE, IRAE, and ultrasonic bath to extract polyphenols from *Centranthus longiflorus* [45]. Their results showed that IRAE required a shorter extraction time and less solvent consumption compared with the other techniques, further confirming that IRAE is a cost-efficient and energy saving technique. This study used water as a solvent, so the phenolic yield obtained was expected to be less than another study where acidified ethanol was used as an extracting solvent, with a TPC value of 14.031 mg GAE/g [46].

In this study, distilled water was used as the extraction solvent, with its boiling point at 100 °C, unlike solvents such as methanol (64.7 °C) and ethanol (78 °C), which have lower boiling points. This allowed the extraction to be conducted at higher temperatures without solvent loss. For the water bath extraction (WBE), the optimal temperature was determined to be 90 °C. In contrast, other studies that used water bath extraction were conducted at lower temperatures for shorter durations, such as one study where extraction was performed at 75 °C for 30 min using pure methanol as the solvent [47]. Another study carried out the extraction overnight at room temperature with methanol, eliminating the need for high temperatures [48]. In the case of WBE, the longer extraction time presents a risk of solvent loss if solvents with lower boiling points are used. However, the results of this study show that infrared-assisted extraction (IRAE) achieved a high polyphenol yield in just 10 min at 80 °C (a tolerable temperature for distilled water) offering significant energy savings due to the shorter extraction time.

### 3.3. Microbiological Activity

#### 3.3.1. Disk Diffusion Assay

The antibacterial activities of WBE and IRAE extracts studied by the disk diffusion method are presented in Table 3. Moreover, the WBE extract showed average inhibition zones of 10, 14, and 10.5 mm against *Listeria monocytogenes*, *Proteus* sp., and *Salmonella* sp., respectively. Regarding the IREA, it inhibited *Listeria monocytogenes*, *Proteus* sp., and *Salmonella* sp. with average inhibition zones of 10, 10, and 10.5 mm, respectively. On the other hand, no inhibition of bacterial growth was detected against the other studied bacterial strains. Similar results were obtained in the study conducted that tested many food by-product extracts for their antibacterial activities, including potato peels, where the results showed that the phenolic extracts exhibited a moderate inhibition against both Gram-negative (*E. coli*) and Gram-positive (*Staphylococcus aureus*) bacterial strains with no effect on *Bacillus cereus* [49].

#### 3.3.2. MIC Results

The phenolic extracts presenting an antibacterial effect against the tested bacterial strains were selected for the determination of the MIC. The results showed that the WBE extract inhibited *Salmonella* sp. isolate at an MIC value of 1.04 mg GAE/g DM with no detected inhibition by the IRAE. On the other hand, *Proteus* sp. and *Listeria monocytogenes* were resistant for WBE and IRAE, and no MIC values were detected. A study evaluated the antibacterial effect of ethanolic phenolic extract, concentrated using a rotary evaporator and turned into powder then diluted in broth to reach a concentration of 5 mg/mL [28]. The bacterial strains tested were *E. coli*, *S. enterica*, *K. pneumonia*, *S. aureus*, and *L. monocytogenes*. For MIC results, *E. coli* was inhibited only in the 5 mg/mL extract, while the two other bacterial strains required a lower concentration 1.25 mg/mL for *S. aureus* and 2.5 mg/mL for *S. enterica*, respectively. It is important to note that the extract was concentrated via a rotary evaporator and used ethanol as an extracting solvent to reach such results. However, in this study, the crude extracts themselves extracted in distilled water showed an inhibitory effect on two Gram-negative (*Salmonella* sp. and *Proteus* sp.) and one Gram-positive (*L. monocytogenes*) bacterial strains. To boost the MIC results, concentrating the extract by using a rotary evaporator or by freeze-drying can facilitate the microbiological testing at different concentrations and determine which concentration can give the better antibacterial effect.

### 3.4. Anti-Sprouting Effect

**Figure 6:**
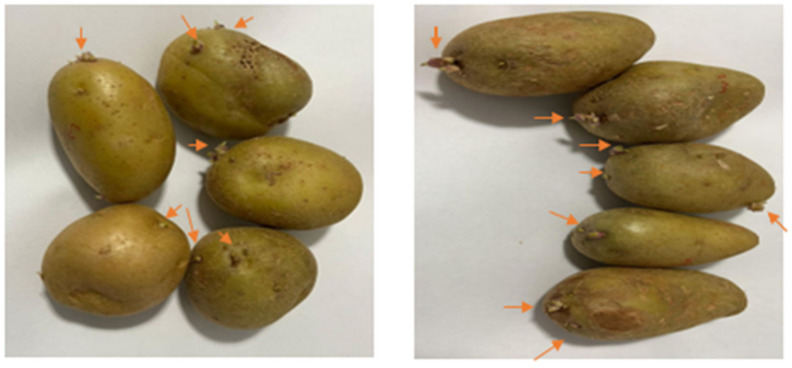
Potato peels extract-treated potatoes (**left**) and water-treated potatoes (**right**) at room temperature at the end of the experiment. Arrows point to the present sprouts.

The potatoes were split into two groups: a control group that was treated with tap water and the experimental group treated with the potato peels extracts, as shown in Figure 6 with the visual sprout appearance. The potato peels extract treated potatoes that were left at RT showed a significant difference in inhibiting the number of sprouts across the twelve days during which the testing ran compared with the control group (*p* < 0.05). Regarding the sprout size, there was a significant difference in the inhibition (*p* < 0.05) for nine days while for the remaining days there was no significance in the inhibition (*p* > 0.05). These results show that potato peels extract has a positive effect on inhibiting the sprouting of potatoes at RT. Sprout number and size results for each day are presented in Table 4.

The results of this study showed a significant inhibition effect at room temperature. It is important to mention that most potatoes are stored at 8–12 °C cold storage temperature in case of long-term storage, where this temperature allows the minimum accumulation of reducing sugars within the potatoes [50]. Reducing sugars are the reason for giving an off flavor and undesirable color change when potatoes are cooked or fried. Even at this cold storage temperature, once the natural dormancy phase of the potatoes is over, sprouting would begin leading to the use of sprout suppressant. The chemical generally used (CIPC), fogged once or twice a year depending on the storage duration, was found to be more effective at a temperature of 15 °C or less, so when the temperature exceeds that, the effect decreases [23]. In our study, the potatoes were exposed to the extract only once and by partially submerging them in the extracts as mentioned previously, and there was a suppression effect at room temperature. So, it is expected that if stored at a colder temperature there would be a prolonged inhibition effect. Therefore, it is important to evaluate the effect that the extract has at different temperatures and by trying the fogging method as used when treating potatoes with CIPC. Compared with the applied chemical sprout inhibitors, the use of potato peels extracts offers economic advantages by utilizing an agricultural by-product, reducing waste disposal costs, and reducing the requirement for synthetic chemical usage. The natural extracts could potentially lower long-term costs by providing an eco-friendly, cost-effective alternative to the traditional chemical treatments.

Additionally, a study evaluated various individual organic compounds and found that maleic acid and L-tartaric acid inhibited sprouting up to six weeks while ferulic, gallic, and caffeic acid inhibited sprouting for 1–2 weeks [51]. The tested concentrations were from 0.1 mg/mL to 0.3 mg/mL. It is important to mention that according to the literature, potato peels present caffeic acid, chlorogenic acid, and its isomers in large amounts, and some references noted that it is the polyphenol present highest in the peels [52]. Chlorogenic acid was not tested in the previously mentioned study nor was a combination of phenolic acids (as is the case with the extract). The combination of the various previously mentioned phenolic acids in potato peels could lead to this inhibition effect on sprouting. To evaluate further, each phenolic acid present in the potato peels extract can be individually tested to further determine what phenolic acids have the highest sprout suppression effect.

### 3.5. Identification and Quantification of Phenolic Compounds Using HPLC

Table 5 represents the polyphenols identified and quantified using HPLC in the potato peels WBE and IRAE. The compounds detected in both extracts were catechin and chlorogenic acid. Protocatechuic acid and gallic acid were detected only in the IRAE extract, while rutin, caffeic acid, and *p*-coumaric acid were detected only in the WBE extract. These results are consistent with the literature on the polyphenol composition of potato peels [46,53,54,55]. Our findings also align with reports identifying catechin and rutin as two of the major flavonoids present in the peels [56]. According to previous studies, the most abundant polyphenols in potato peels are caffeic acid and chlorogenic acid, which is consistent with our findings [52,57]. Notably, catechin detected in the IRAE extract (0.12 mg/g DM) was higher than in the WBE extract (0.05 mg/g DM), and similarly, chlorogenic acid was more abundant in the IRAE extract (0.02 mg/g DM) compared with the WBE extract (0.005 mg/g DM). The HPLC results indicate that phenolic compound extraction is influenced by the specific characteristics of each compound and the type of solvent used [58]. Since distilled water was the extracting solvent in this study, the extracted phenolic compounds were lower in quantity than those reported in the literature. For example, one study found 0.963 mg/g dry weight of chlorogenic acid using 80% methanol as the solvent, whereas our findings detected 0.02 mg/g DM of chlorogenic acid in the IRAE extract [52].

## 4. Conclusions

This study focused on the valorization of potato peels through the extraction of bioactive compounds. The yield of phenolic compounds was influenced by the extraction time, temperature, and method employed. Optimal conditions varied between techniques, with IRAE requiring lower temperatures and significantly shorter durations than WBE to achieve similar yields. For WBE, the optimum conditions were 90 °C for 70 min, yielding 3.5 mg GAE/g DM, while for IRAE, optimal extraction was achieved at 80 °C for just 10 min, yielding the same number of polyphenols. This demonstrates IRAE’s potential for reducing energy costs compared with WBE.

The extracts were also evaluated for antibacterial activity using the agar disk diffusion method, demonstrating inhibitory effects on three bacterial strains: one Gram-positive (*L. monocytogenes*) and two Gram-negative (*Salmonella* sp. and *Proteus* sp.), with the WBE extract showing an MIC of 1.04 mg GAE/g DM against *Salmonella* sp.

In addition, this study is the first of its kind to investigate the sprout-inhibiting properties of potato peels extracts. Indeed, the extracts exhibited significant anti-sprouting effects on potatoes, all while utilizing distilled water as a safe and environmentally friendly solvent. This study concludes that potato peels extracts are a valuable source of bioactive compounds with potential applications in the food industry as natural preservatives or antioxidants, as well as a promising anti-sprouting agent for potatoes.

## 5. Patent

El Darra, N., Helmi, L., Rajha, H.N., Debs, E., Louka, N. “Potato sprouting inhibition using potato peels extract”. Lebanese patent number 2022/12-12702L granted on 22 December 2022.

## Figures and Tables

**Table 2:** Regression equations and R^2^ values of TPC and DPPH for each extraction technique.

WBE	IRAE
R^2^ (TPC) = 95.16% TPC = 0.927 + 0.00137 × T − 0.00495 × t + 0.000016 × T^2^ + 0.00061 × T × t − 0.00022 × t^2^	R^2^ (TPC) = 90.98% TPC = −1.704 + 0.0296 × T + 0.084 × t + 0.00032 × T^2^ − 0.00098 × T × t − 0.000237 × t^2^
R^2^ (DPPH) = 91.48% DPPH = 328 − 11.84 × T − 0.64 × t + 0.12 × T^2^ − 0.00165 × T × t + 0.0125 × t^2^	R^2^ (DPPH) = 91.53% DPPH = 267 − 12.1 × T + 1.38 × t + 0.136 × T^2^ − 0.012 × T × t − 0.00698457 × t^2^

**Table 3:** Average inhibition zones of IRAE, WBE, and positive control against the tested bacterial strains.

Bacterial Strains	IRAE Disk	WBE Disk	Gentamicin Disk (10 μg)
*Listeria monocytogenes*	10 mm	10 mm	37 mm
*Proteus* sp.	10 mm	14 mm	25 mm
*Salmonella* sp.	10.5 mm	10.5 mm	32 mm

**Table 4:** Results of sprout number and sprout size results at room temperature (W = water, E = extract).

	Day 1	Day 2	Day 3	Day 4	Day 5	Day 6	Day 7	Day 8	Day 9	Day 10	Day 11	Day 12
Total sprout number W	4	14	23	32	43	50	62	65	70	71	76	76
Total sprout number E	0	1	5	10	14	23	26	26	29	32	36	41
Total sprout size (cm) W	0.2	1.2	2.55	4.2	7.65	11.85	14.05	15.6	19.45	20.95	24.05	26.3
Total sprout size (cm) E	0	0.1	0.3	0.75	1.95	3.65	5.05	5.85	6.95	9.25	11.4	15

**Table 5:** Phenolic compounds concentration (mg/g DM) in potato peels IRAE and WBE. (ND: not detected).

	Rutin	Catechin	Caffeic Acid	Protocatechuic Acid	Chlorogenic Acid	*p*-Coumaric Acid	Gallic Acid
IRAE	ND	0.12	ND	0.019	0.02	ND	0.007
WBE	0.053	0.05	0.119	ND	0.005	0.026	ND

## Data Availability

The original contributions presented in the study are included in the article, further inquiries can be directed to the corresponding author.
